# Fluorescence guided surgery and tracer-dose, fact or fiction?

**DOI:** 10.1007/s00259-016-3372-y

**Published:** 2016-03-28

**Authors:** Gijs H. KleinJan, Anton Bunschoten, Nynke S. van den Berg, Renato A. Valdès Olmos, W. Martin C. Klop, Simon Horenblas, Henk G. van der Poel, Hans-Jürgen Wester, Fijs W. B. van Leeuwen

**Affiliations:** 1 Interventional Molecular Imaging Laboratory, Department of Radiology, C2-S zone, Leiden University Medical Center, Albinusdreef 2, 2300 RC Leiden, The Netherlands; 2 Department of Nuclear Medicine, The Netherlands Cancer Institute – Antoni van Leeuwenhoek Hospital, Plesmanlaan 121, 1066 CX Amsterdam, The Netherlands; 3 Department of Urology, The Netherlands Cancer Institute – Antoni van Leeuwenhoek Hospital, Plesmanlaan 121, 1066 CX Amsterdam, The Netherlands; 4 Department of Head and Neck Surgery and Oncology, The Netherlands Cancer Institute – Antoni van Leeuwenhoek Hospital, Plesmanlaan 121, 1066 CX Amsterdam, The Netherlands; 5 Pharmaceutical Radiochemistry, Technical University Munich, Walther-Meißner-Str. 3, 85748 Garching, Germany

**Keywords:** Sentinel node, SPECT/CT, Multimodal, Fluorescence-guided surgery, Microdosing

## Abstract

**Introduction:**

Fluorescence guidance is an upcoming methodology to improve surgical accuracy. Challenging herein is the identification of the minimum dose at which the tracer can be detected with a clinical-grade fluorescence camera. Using a hybrid tracer such as indocyanine green (ICG)-^99m^Tc-nanocolloid, it has become possible to determine the accumulation of tracer and correlate this to intraoperative fluorescence-based identification rates. In the current study, we determined the lower detection limit of tracer at which intraoperative fluorescence guidance was still feasible.

**Methods:**

Size exclusion chromatography (SEC) provided a laboratory set-up to analyze the chemical content and to simulate the migratory behavior of ICG-nanocolloid in tissue. Tracer accumulation and intraoperative fluorescence detection findings were derived from a retrospective analysis of 20 head-and-neck melanoma patients, 40 penile and 20 prostate cancer patients scheduled for sentinel node (SN) biopsy using ICG-^99m^Tc-nanocolloid. In these patients, following tracer injection, single photon emission computed tomography fused with computed tomography (SPECT/CT) was used to identify the SN(s). The percentage injected dose (% ID), the amount of ICG (in nmol), and the concentration of ICG in the SNs (in μM) was assessed for SNs detected on SPECT/CT and correlated with the intraoperative fluorescence imaging findings.

**Results:**

SEC determined that in the hybrid tracer formulation, 41 % (standard deviation: 12 %) of ICG was present in nanocolloid-bound form. In the SNs detected using fluorescence guidance a median of 0.88 % ID was present, compared to a median of 0.25 % ID in the non-fluorescent SNs (*p*-value < 0.001). The % ID values could be correlated to the amount ICG in a SN (range: 0.003–10.8 nmol) and the concentration of ICG in a SN (range: 0.006–64.6 μM).

**Discussion:**

The ability to provide intraoperative fluorescence guidance is dependent on the amount and concentration of the fluorescent dye accumulated in the lesion(s) of interest. Our findings indicate that intraoperative fluorescence detection with ICG is possible above a μM concentration.

## Introduction

(Near-infrared) fluorescence guidance was introduced to improve the intraoperative identification of lesions and to guide resection of these lesions [1]. This technique has been extensively applied in Japan and provides a high spatial resolution (down to the sub-micrometer range when dedicated microscopes are used) [2]. Superficially in tissue, it offers an increased level of detail compared to the more traditional interventional molecular imaging approaches that are based on radioactive tracers (centimeter range resolution) [3]. Fluorescence guidance technologies are limited by the strong attenuation of the signal, meaning the technology loses value with increasing depth.

To allow fluorescence imaging during the operation, the fluorescent signal, which directly corresponds to the amount of accumulated tracer, has to exceed the detection limit of the imaging system used [4]. To make sure this limit is not an issue, fluorescent dyes are commonly administered to patients at high doses, for example indocyanine green (ICG; administered up to 25 mg) [5], fluorescein (administered up to 500 mg), or methylene blue (administered up to 10 mg; this compound comes with an FDA warning) [6]. When attached to a targeting moiety, the reported doses can still be considered high. Examples are 0.3 mg/kg folate-FITC [7], GE-137 up to 0.18 mg/kg [8], and cetuximab-CW800 (up to 62.5 mg/m^2^) [9]; in a man weighing 75 kg, 62.5 mg/m^2^ corresponds to a total dose of approximately 125 mg (1.7 mg/kg). These latter groups of targeting compounds, therefore, will come at much higher production costs, especially when based on antibodies. The key question that needs to be addressed here is at what minimum dose can fluorescent dyes be used for surgical guidance? The answer to this question, in combination with knowing the binding efficiency of the fluorescence tracer to its target, helps to determine if future fluorescent probes can be can be applied in patients using much lower quantities.

The dose applied to a patient drives the ease of translation and the type of toxicity studies that are required. As a result of the (in-depth) detection sensitivity of nuclear medicine-based modalities, the relation between the administered radiotracer dose and e.g. tumor uptake and in vivo kinetics can be studied in detail. Moreover, because of their high specificity, resulting in efficient accumulation at the site of interest and providing high signal-to-background ratios, imaging can be realized in a microdosing regime. In microdosing studies <100 µg of a new drug or imaging agent can be used in a single patient [10], and extensive expensive toxicity studies can be circumvented. Yet, this is not applied for fluorescence tracers, where in the most first-in-human studies, trial-and-error or blood value evaluations have been used for dose optimization [8]. The microdosing principle is, in part, also driven by economical aspects; the administered dose will directly relate to costs and thus is of importance for both business models and future reimbursement. For routine use of targeted fluorescence tracers in patients rather than first-in-human trials, it is, therefore, crucial to determine the lowest possible dose at which this technology can be applied.

Hybrid tracers, which contain both a radioactive and a fluorescent signature, may help to provide insight in the dose required for efficient intraoperative detection of a fluorescent signature. Based on the radioactive signal of such tracers, combined with nuclear imaging, a quantitative evaluation of the biodistribution can be made. This, in turn, can be directly related to the intraoperative fluorescence-based detection rate. A number of hybrid tracers have been studied in humans [3], and of these ICG-^99m^Tc-nanocolloid has been most extensively used [11–13]. This tracer was introduced to complement preoperative sentinel node (SN) mapping and intraoperative radioactivity-based SN localization with intraoperative near-infrared fluorescence guidance [11–13]. Because of inter-patient variations in lymphatic drainage or differences in tissue composition, e.g. obesity, in some cases SNs do not contain tracer, or tracer-containing nodes cannot be detected intraoperatively, respectively. Nevertheless, the SNs defined at single photon emission computed tomography combined with computed tomography ( SPECT/CT) display a radioactive and fluorescent signal after more accurate ex vivo evaluation [11–13]. We thus reasoned that via assessment of the radioactive signature of ICG-^99m^Tc-nanocolloid, it should be possible to determine the lower detection limit at which fluorescence guidance was still feasible intraoperatively. To adequately evaluate these aspects, malignancies with both superficial (head-and-neck melanoma, penile cancer; fluorescence camera for open surgery) and deep (prostate cancer; fluorescence laparoscope) lymphatic drainage were included.

## Methods

### Preparation of the hybrid tracer

For penile cancer patients and head-and-neck melanoma patients, ICG-^99m^Tc-nanocolloid was prepared as previously described [12, 13]. In short, a vial of nanocolloid (GE Healthcare, Leiderdorp, the Netherlands) (0.5 mg human serum albumin (HSA)) was dissolved in 2 mL saline (0.9 g/L NaCl) containing freshly eluted pertechnetate (1400 MBq) resulting in an HSA concentration of 3.76 μM. A vial of ICG (25 mg) (Pulsion Medical Systems, Feldkirchen, Germany) was dissolved in 5 mL water for injection. 50 μL of this ICG solution was added to the ^99m^Tc-nanocolloid resulting in an ICG concentration of 161 μM. From this vial approximately 90 MBq ± 10 % was subtracted and supplemented to 0.4 mL with saline. This solution contained 0.49 nmol (32 μg) HSA and 20.8 nmol (16 μg) ICG.

For prostate cancer patients two different tracer formulations were evaluated [11]. The first group received the ICG-^99m^Tc-nanocolloid prepared according to an earlier used protocol. In this protocol a vial of nanocolloid (0.5 mg HSA) was dissolved in 1 mL saline containing freshly eluted pertechnetate (700 MBq) resulting in an HSA concentration of 7.52 μM. A vial of ICG (25 mg) was dissolved in 5 mL water for injection. Of this ICG solution, 50 μL was added to the ^99m^Tc-nanocolloid resulting in an ICG concentration of 323 μM. From this vial approximately 250 MBq ± 10 % (0.4 mL) was injected. This solution contained 3.01 nmol (200 μg) HSA and 129 nmol (100 μg) ICG.

The second group received ICG-^99m^Tc-nanocolloid prepared according to a new protocol. In this protocol a vial of nanocolloid (0.5 mg HSA) was dissolved in 2 mL saline containing freshly eluted pertechnetate (300 MBq) resulting in an HSA concentration of 3.76 μM. A vial of ICG (25 mg) was dissolved in 5 mL water for injection. 50 μL of this ICG solution was added to the ^99m^Tc-nanocolloid resulting in an ICG concentration of 161 μM. The complete content of the prepared vial was injected. This solution contained 7.52 nmol (500 μg) HSA and 323 nmol (250 μg) ICG.

All tracer preparations were performed under good manufacturing practice and under supervision of the institution’s pharmacist.

### Size exclusion chromatography

Size exclusion chromatography (SEC) provided a laboratory set-up able to simulate the migratory behavior of ICG-nanocolloid in tissue. The small diameter pores in the stationary phase mimic the extracellular space in tissue.

ICG-nanocolloid was prepared as described above for the head-and-neck melanoma and penile cancer and the new formulation used in prostate cancer patients (161 μM ICG), but without the radioactive pertechnetate addition. Samples of 450 µL were extracted from the vial at 2, 4, 6, and 8 h after preparation. From each sample, 450 µL was loaded onto a size exclusion column (52 × 9.6 mm) containing Sephadex G-50 fine (Sigma-Aldrich, St. Louis, Missouri, USA). Gravity was used to elute the column with saline.

Six fractions of 500 μL were collected over approximately 20 min and diluted with 2 μM HSA to obtain light absorption below 0.3 at 803 nm. Absorption of each fraction was measured with a Lambda Bio 20 UV–Vis spectrometer (Perkin Elmer, Waltham, MA, USA) and the fraction of the total ICG that co-eluted with nanocolloid in these six fractions was calculated [14].

### Absorption

To determine the presence of different stacked forms of ICG next to the fluorescent monomer form, UV–Vis absorption spectra were obtained of the different SEC fractions. Samples containing the clinical concentration of ICG (161 μM) were prepared by dissolving ICG in water for injection (5 mg/mL) and subsequent dilution of 50 μL of this stock in 2 mL water for injection, saline, or in a vial of nanocolloid dissolved in 2 mL saline. UV–Vis absorption spectra were recorded in a 1 mm optical pathway quartz cuvette.

### Clinical study

#### Patients

This retrospective analysis evaluated the fluorescence detection of SNs in patients that underwent SN biopsy using the hybrid tracer ICG-^99m^Tc-nanocolloid: 20 patients with melanoma in the head-and-neck area, 40 patients with penile cancer, and another 20 patients with prostate cancer were included. From all patients written informed consent was obtained.

The surgical procedures in melanoma patients and penile cancer patients were open procedures. The prostatectomies for prostate cancer were robot-assisted laparoscopic procedures. All procedures were performed between December 2010 and June 2013.

Given the intention to identify the lower detection levels for intraoperative fluorescence guidance, the selection of patients for this study was biased towards patients with intraoperative non-visualization; patients where fluorescence detection failed during surgery were selectively included.

#### Injection of the hybrid tracer and preoperative nuclear imaging

Tracer injection and preoperative SN mapping for head-and-neck melanoma, penile cancer and prostate cancer have been described previously [11–13].

In all patient groups image acquisition was performed with a SPECT/CT dual-head gamma camera (SymbiaT; Siemens, Erlangen, Germany) equipped with parallel low energy high resolution collimators. The following configurations were used during preoperative imaging: planar lymphoscintigraphy was acquired at source-to-collimator distances up to 15 cm with an image acquisition time of 5 min. Non-circular SPECT (256 × 256 matrix, 40 frames, 30 s/frame) was performed in combination with a low-dose CT (130 kV, 40mAs, B30s kernel). Total acquisition time: approximately 14 min including the low - dose CT. The low-dose CT was based on 5 mm slices in penile cancer patients, and on 2 mm slices in head-and-neck melanoma and prostate cancer patients.

When the circumferential borders of the SNs could be clearly determined on low-dose CT, the volume of the SNs was determined. Diameters (a = ½ x length, b = ½ x width) of the SN were measured on the low-dose CT, in cms, and the volume was calculated according to the following formula:

$$ V\left(c{m}^3\right)=\frac{4}{3}\uppi \times \left(\mathrm{a}\right)\times \left(\mathrm{b}\right)\times \left(\mathrm{b}\right) $$

#### Image analysis

OsiriX medical imaging software (Pixmeo, Geneva, Switzerland) was used to measure total pixel values [15]. Measurements of the injection site and SNs were performed on the SPECT component of the SPECT/CT scan using a 3D region-growing tool with calculation of the pixel values. Besides the injection site, and the SN(s), the rest activity (higher-echelon nodes, spillage) was calculated. All calculated pixel values were combined resulting in the total pixel value on SPECT/CT.

Liver uptake as a result of shunting during the tracer administration was not taken into account because it could not be accurately determined in all patients; in the head-and-neck and penile cancer patients, the liver was not present in the field of view when performing lymphoscintigraphy and SPECT/CT imaging, and in the prostate cancer patients the liver was only partly present in the field of view.

Based on the measured total pixel values in the SPECT scan the percentage of drainage of the injected dose to an individual SN was calculated using the following formula:

$$ Drainage\kern0.20em to\kern.20em SN\kern.20em \left(\%\right)=\frac{Total\kern.20em  pixel\kern.20em  value\kern.20em  in\kern.20em  an\kern.20em  in dividual\kern.20em SN}{Total\kern.20em  pixel\kern.20em  value\kern.20em  on\kern.20em  SPECT}\times 100\% $$

#### Determination nodal ICG accumulation

The SNs were divided into four groups: nodes having received drainage of 0–1, 1–2, 2–3, and >3 % of the total injected dose (ID). Based on the known ratio of ICG and ^99m^Tc (see above) the percentage of ^99m^Tc drainage determined on SPECT was used to calculate the amount of ICG that accumulated in the SN using the following formula:

$$ ICG\kern.20em in\kern.20em SN\kern.20em (nmol)=Drainage\kern.20em to\kern.20em SN\kern0.20em \left(\%\right)\times Injected\kern.20em dose\kern.20em ICG\kern0.20em (nmol)\times Percentage\kern.20em of\kern.20em nanocolloid\kern0.20em bound\kern0.20em ICG\kern.20em \left(\%\right) $$

Here the injected dose of ICG was 20.8 nmol for the penile cancer and head-and-neck melanoma patients, 129 nmol for the old prostate preparation, and 323 nmol for the new prostate preparation. The percentage of ICG bound to nanocolloid after mixing the two was determined using SEC (see Results).

To determine the concentration of ICG in the SNs, the uptake of ICG in the SNs was divided by the volume of the SN (calculated as stated above) using the following formula:

$$ \left[ICG\right]\  in\ SN\ (nM)=\frac{ICG\  in\ SN\ (nmol)}{Volume\  of\ SN\ \left(c{m}^3\right)}\times 1000 $$

#### Fluorescence detection during surgical procedure

For open procedures (penile cancer, head-and-neck melanoma) the PhotoDynamicEye (Hamamatsu Photonics KK, Hamamatsu, Japan) was used as described previously [12, 13].

SN biopsy combined with extended pelvic lymph node dissection and prostatectomy was performed robot-assisted (da Vinci S, Intuitive, Sunnyvale, CA, USA) in prostate cancer patients. For fluorescence detection in the laparoscopic robotic setting an additional fluorescence laparoscope (KARL STORZ GmbH & Co. KG, Tuttlingen, Germany) was used [11].

For localization of the SNs, intermittently the gamma probe and fluorescence camera were used. To limit the influence of tissue-based signal attenuation, fluorescence-based visibility of the SNs (yes/no) was assessed when they were maximally exposed by the surgeon. This yielded a portion of SNs that could be resected using intraoperative fluorescence guidance as well as a portion SNs that contained not enough signal to allow for intraoperative fluorescence detection.

#### Statistical analysis

Statistical Package for the Social Sciences (SPSS, IBM, version 22) was used to calculate for continuous variables, the mean or median and interquartile range (IQR; 25–75 %) and range. For discrete variables, frequencies and percentages are reported. A Mann–Whitney U test was performed to compare the means of uptake (% ID) of fluorescent and non-fluorescent SNs and to compare the means of uptake (% ID) of tumor-negative and tumor-positive SNs. A comparison of the fluorescence detection rate between tumor-negative SNs and tumor-positive SNs was performed with a Fisher’s exact test.

#### Pathology

(Histo-)pathological evaluation of the SNs was performed as previously described [11–13]. The diameters of the excised SNs were not defined in the pathological specimens, merely the tumor status and size of metastases.

## Results

### Size exclusion chromatography

Preparation of the hybrid tracer ICG-^99m^Tc-nanocolloid, resulted in an ICG to HSA ratio of approximately 43 to 1. ICG is well soluble in water, but poorly soluble in saline. This poor solubility in combination with the tendency of HSA to bind hydrophobic, aromatic, and fatty acid like compounds, led to the formation of a non-covalent complex between nanocolloid and ICG [16, 17].

**Fig. 1:**
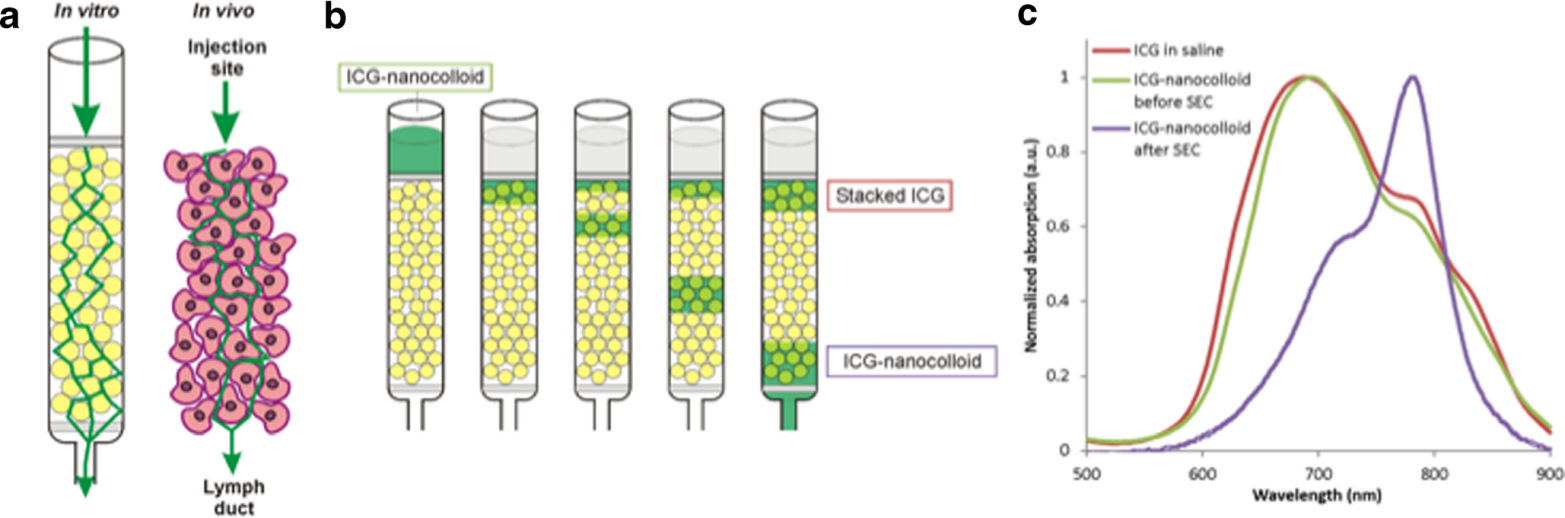
Schematic overview of the size exclusion chromatography experiments. Phantom set-up to mimic the in vivo drainage of the hybrid tracer (**a**). Size exclusion chromatography of the prepared hybrid tracer results in an ICG-nanocolloid fraction and a fraction of stacked ICG that accumulated on the column (**b**). Absorption spectra of ICG dissolved in saline, and of ICG-nanocolloid before and after size exclusion chromatography (**c**)

When ICG-^99m^Tc-nanocolloid is injected in or around the primary tumor it ends up in the extra-cellular space, hence the marking of the injection site [18]. From there it will move, due to the increased interstitial pressure, through the spaces between the cells and through the extracellular matrix into the lymphatic ducts (Fig. 1a). A phantom set-up for this process was provided by SEC chromatography; the columns used in SEC consist of small particles (with interstitial spaces) that allow gravity dependent flow wherein different sized compounds are eluted at different speeds. In this set up the large ICG-nanocolloid particles eluted first. 41 % (standard deviation: 12 %) of ICG was present in this fraction, corresponding to 17 ICG molecules per HSA protein, which is in close agreement with previous findings [14]. The ICG-nanocolloid fractions remained stable for at least 8 h after preparation. The SEC experiment also demonstrated that the residual “optically silent” ICG fraction remained present on the column indicating a small size as well as poor solubility. Only when the column was washed extensively with demineralized water or a HSA solution did this fraction elute from the column. We assume similar behavior for the injected formulation at the site of injection.

### Spectral analysis

ICG diluted in saline (161 μM) without the presence of nanocolloid showed a strong and broad absorption peak around 685 nm which is associated with non-fluorescent (“optically silent”) H-stacking, J-stacking, and more elaborate stacking forms of ICG (Fig. 1c) [19]. The non-separated ICG-nanocolloid formulation gave a similar absorption spectrum (Fig. 1c), indicating that the solution contained a fair amount of stacked ICG. The ICG-nanocolloid fraction of the SEC purification, however, displayed an absorption peak correlating to the fluorescent monomer form of ICG (Fig. 1b and c).

### Preoperative imaging

**Fig. 2:**
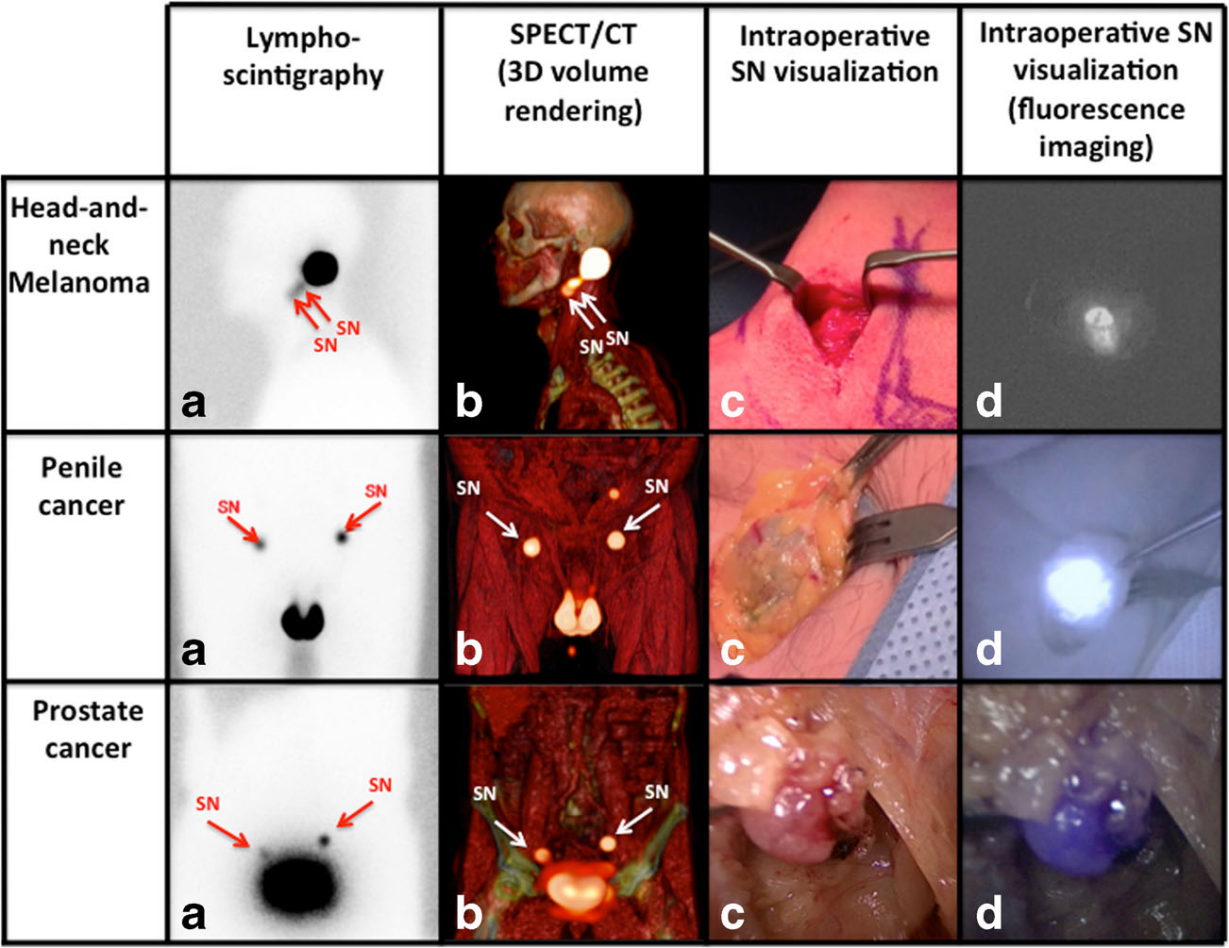
Typical examples of the sentinel node biopsy procedure when using the hybrid approach. In the first row a head-and-neck melanoma case is illustrated, in the second row a penile cancer case is illustrated and in the third row a prostate cancer case is illustrated. From *left* to *right* **a**) lymphoscintigram with the location of the SN(s) (*arrows*); **b**) a 3D volume rendering of the SPECT/CT (*arrows*); **c**) white light imaging of the SN in vivo; and **d**) in vivo fluorescence imaging of the SN. Fluorescence imaging with the PDE generates a *black-and-white* image, the fluorescence laparoscope shows the fluorescence signal in the SN in *blue*

In our clinical dose-evaluation studies we focused on three patient groups, namely patients with head-and-neck melanoma (*n* = 20; 60 SNs), penile cancer (*n* = 40; 104 SNs) and prostate cancer (*n* = 20; 65 SNs). Figure 2 provides typical examples of the pre- and intraoperative imaging findings when using the hybrid tracer for SN biopsy [11–13].

### Intraoperative fluorescence guidance versus hybrid tracer uptake in the sentinel node(s)

A total of 229 SNs were removed during surgery of which 203 SNs (88.6 %) were identified intraoperatively via their fluorescence signature. The details for the three patient groups are further specified in Table 1. It must be noted, that in contrast to our previously published studies [11–13], based on the biased selection criteria, this study reports a higher non-visualization rate during the operation. In the overall group, ex vivo analysis allowed visualization of 97.8 % (224/229) of the SNs via fluorescence imaging. In some cases the small SNs with small amounts of tracer were embedded in fatty tissue which prevented their identification even ex vivo.

**Table 1:** Drainage % hybrid tracer in sentinel nodes related to the amount of ICG (nmol)

Percentage injected dose that drained to the SN	Estimated amount ICG (nmol)	Percentage successful surgical identification through fluorescence guidance (SN-based)	Percentage of SNs that were missed intraoperatively using fluorescence guidance (SN-based)
Head-and-neck melanoma patients (60 SNs)			
0–1 % ID	0–0.085	80.0 % (32/40)	89 % (8/9)
1–2 % ID	0.085–0.17	92.4 % (12/13)	11 % (1/9)
2–3 % ID	0.17–0.255	100 % (6/6)	0 %
>3 % ID	>0.255	100 % (1/1)	0 %
Penile cancer patients (104 SNs)			
0–1 % ID	0–0.085	85.1 % (40/47)	88 % (7/8)
1–2 % ID	0.085–0.17	94.8 % (18/19)	12 % (1/8)
2–3 % ID	0.17–0.255	100 % (17/17)	0 %
>3 % ID	>0.255	100 % (21/21)	0 %
Prostate cancer patients (10 SNs; old preparation)			
0–1 % ID	0–0.53	100 % (7/7)	0 %
1–2 % ID	0.53–1.06	100 % (1/1)	0 %
2–3 % ID	1.06–1.59	0 % (0/1)	100 % (1/1)
>3 % ID	>1.59	100 % (1/1)	0 %
Prostate cancer patients (55 SNs; new preparation)			
0–1 % ID	0–1.32	81.1 % (30/37)	88 % (7/8)
1–2 % ID	1.32–2.65	88.9 % (8/9)	12 % (1/8)
2–3 % ID	2.65–3.97	100 % (2/2)	0 %
> % ID	>3.97	100 % (7/7)	0 %

SNs were categorized with respect to the % ID uptake in the SN as defined on preoperative SPECT imaging: 0–1, 1–2, 2–3, and >3 %; of the overall group, 57.2 % of the SNs was categorized in the 0–1 % group (Table 1). In the overall group, the median hybrid tracer uptake of a via fluorescence imaging identified SN was 0.88 % ID (IQR 0.25–2.22 % ID), which was significantly higher than the median hybrid tracer uptake in an SN that could not be detected in vivo using fluorescence imaging, which was 0.25 % ID (IQR 0.13–0.73 % ID) (Mann–Whitney U test, *p*-value < 0.001). In the 0–1 % ID drainage range intraoperative fluorescence guidance was least efficient (Table 1).

The open surgical procedures consisted of two groups. In the head-and-neck melanoma group, SNs that could be identified intraoperatively using fluorescence guidance showed a median hybrid tracer uptake of 0.33 % ID (IQR 0.13–1.31 % ID) (Table 2). The SNs that could not be identified intraoperatively using fluorescence guidance showed a median hybrid tracer uptake of 0.14 % ID (IQR 0.12–0.44 % ID). For the penile cancer patient group these values were 1.37 % ID (IQR 0.63–2.86 % ID) and 0.38 % ID (IQR 0.13–0.94 % ID), respectively (Table 2).

**Table 2:** Percentage uptake in sentinel nodes related to the possibility of measurement of sentinel nodes on CT and to intraoperative fluorescence detection

Fluorescent	SNs measured on low dose CT	Median % ID (IQR)	Median estimated nmol ICG per SN (IQR)	Median SN volume (cm^3^) (IQR)	Median estimated concentration ICG in SN μM (IQR)
Head-and-neck melanoma					
Yes	Yes (*n* = 33)	0.37 (0.13–1.67)	0.031 (0.011–0.142)	0.11 (0.03–0.23)	0.39 (0.15–0.91)
	No^*^ (*n* = 18)	0.33 (0.15–1.10)	0.028 (0.012–0.094)	–	
No	Yes (*n* = 6)	0.23 (0.08–0.72)	0.020 (0.007–0.061)	0.03 (0.01–0.043)	1.32 (0.28–3.24)
	No* (*n* = 3)	0.14 (0.136)	0.012 (0.012)	–	
Penile cancer					
Yes	Yes (*n* = 90)	1.52 (0.70–2.90)	0.129 (0.060–0.247)	0.57 (0.36–1.12)	0.24 (0.08–0.48)
	No* (*n* = 6)	0.55 (0.20–1.07)	0.047 (0.017–0.910)		
No	Yes (*n* = 8)	0.38 (0.13–0.94)	0.032 (0.011–0.080)	0.64 (0.47–1.30)	0.08 (0.019–0.24)
	No* (*n* = 0)	–	–	–	
Prostate cancer, old preparation					
Yes	Yes (*n* = 5)	0.57 (0.44–2.10)	0.048 (0.037–0.179)	0.17 (0.08–0.86)	1.74 (1.01–4.52)
	No* (*n* = 4)	0.17 (0.07–0.87)	0.090 (0.037–0.46)	–	
No	Yes (*n* = 1)	2.30 (2.30)	1.22 (1.22)	0.24	5.16
	No* (*n* = 0)	–	–	–	
Prostate cancer, new preparation					
Yes	Yes (*n* = 36)	0.55 (0.14–1.34)	0.417 (0.185–1.769)	0.17 (0.09–0.32)	2.86 (1.27–15.62)
	No* (*n* = 11)	0.87 (0.63–3.15)	1.148 (0.832–4.158)	–	
No	Yes (*n* = 5)	0.29 (0.14–0.54)	0.383 (0.184–0.71)	0.14 (0.09–0.23)	2.44 (1.79–3.87)
	No* (*n* = 3)	0.08	0.106	–	

* The inability to perform the required volumetric measurements in the low-dose CT depended mostly on the size of the SNs and the poor quality of the images. *CT* computed tomography, *ID* injected dose, *IQR* interquartile range, *SN* sentinel node, *ICG* indocyanine green

For the robot-assisted laparoscopic surgery application, in the first group (old tracer preparation [11]), the SNs that could be identified with fluorescence imaging (9/10 SN) had a median uptake of 0.56 % ID per SN (IQR 0.17–0.83 % ID), only one SN could not be identified, giving a somewhat high % ID, with intraoperative fluorescence imaging (2.30 % ID) (Table 2). In the second group (new tracer preparation), which received ICG-^99m^Tc-nanocolloid in a high dose formulation, the SNs identified with fluorescence imaging (*n* = 47) had a median of 0.70 % ID uptake (IQR 0.18–1.45 % ID) versus 0.23 % ID (IQR 0.09–0.60 % ID) in the SNs that could not be identified with fluorescence imaging SN (*n* = 8) (Table 2).

It must be noted that the above-mentioned calculations are likely to overestimate the % ID in the SN and as such the amount of ICG present in the node. This is a direct consequence of the inability to measure the (3D) pixel values in the liver resulting in an underestimation of the total counts measured in the SPECT/CT images.

### Volumes of the sentinel nodes

The concentration of the hybrid tracer in a SN, and thus the concentration of ICG, was determined by relating the amount of ICG in a SN to the volume of that SN derived from the SPECT/CT images (Table 2). For the SNs located in the neck, the median size was 0.072 cm^3^ (IQR 0.033–0.18 cm^3^). This value deviates from the 0.50 cm^3^ and an IQR of 0.36–0.85 cm^3^ that was previously reported [20]. A reason for this difference could be that the size of lymph nodes is very much dependent on the neck level where they are located [21]. In the group of penile cancer patients a median of 0.57 cm^3^ (IQR 0.38–1.12 cm^3^) was found. In the prostate cancer group, the measured SNs (*n* = 47) had a median volume of 0.17 cm^3^ (IQR 0.09–0.27 cm^3^). The size of the nodes in the penile cancer group and prostate cancer groups were comparable with the sizes reported in previous literature of lymph nodes in the inguinal and pelvic area [22, 23]. The SNs that could not be measured on CT, overall had a lower % ID uptake of tracer, suggesting they were small in size.

### Amount of ICG in quantity and concentration in relation to intraoperative fluorescence-based detectability

**Fig. 3:**
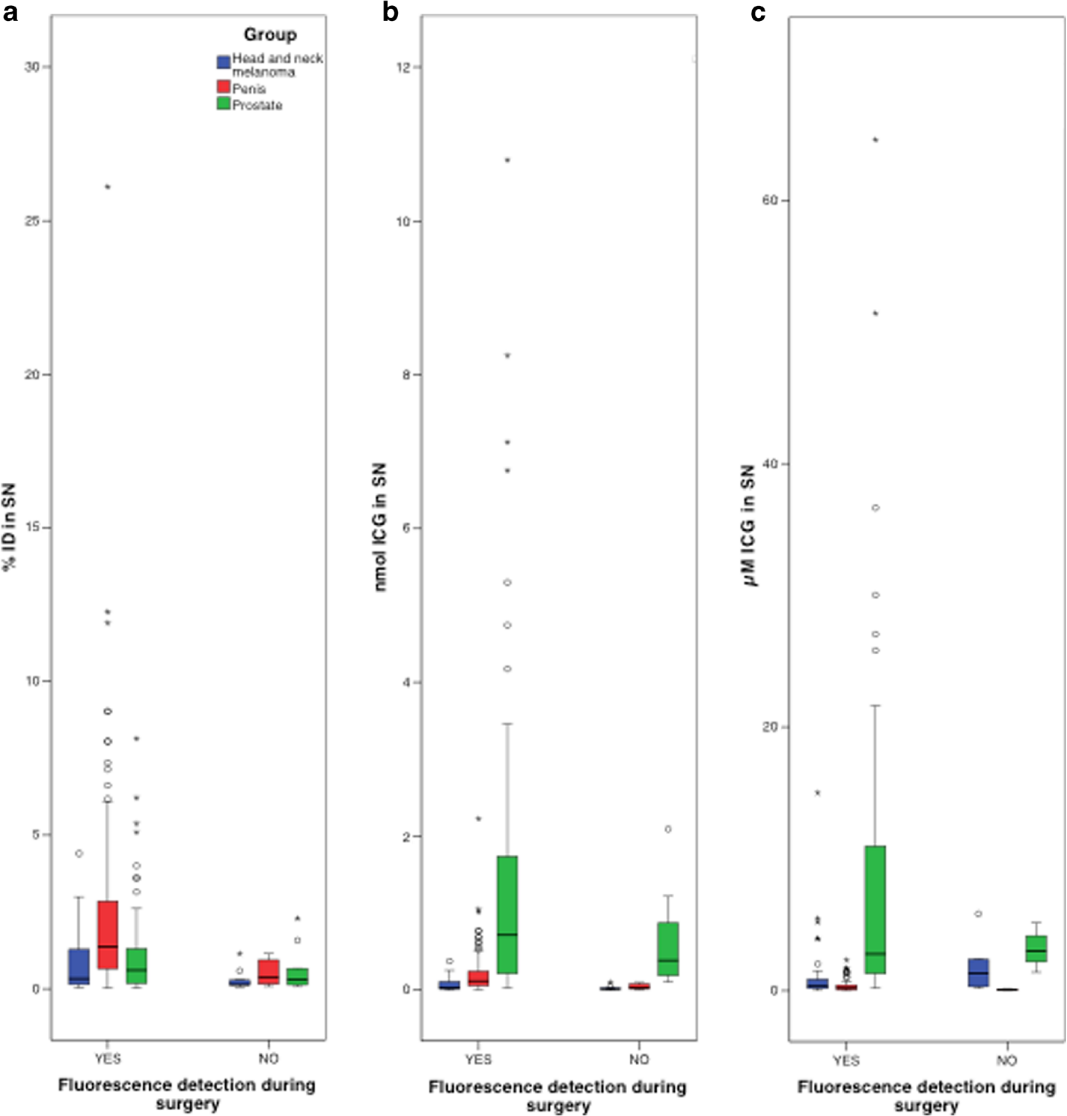
Boxplots of intraoperative fluorescence detection versus % ID, amount of ICG in nmol and the concentration indocyanine green in the sentinel nodes. **a** % ID in relation to the intraoperative fluorescence detection; **b** The amount of ICG (nmol) in relation to the intraoperative fluorescence detection; and **c** The concentration of ICG (μM) in relation to the fluorescence detection

A clear relation was found between the detection rate of the SNs by fluorescence imaging and the hybrid tracer uptake (in % ID and amount of ICG; Fig. 3a and b). However, when the detection rate was related to the ICG concentration, defined using low-dose CT-derived SN volumes, the relation was not clear (Fig. 3c).

In head-and-neck melanoma, with the smallest SNs, the concentration of ICG in the SNs that were detected by fluorescence imaging was similar compared to the SNs that were not detected by fluorescence imaging (median 0.39 μM vs. 1.32 μM; Table 2 and Fig. 3). In penile cancer patients the difference was more distinct, showing a higher ICG concentration in SNs that could be detected intraoperatively compared to those that could not be detected intraoperatively via fluorescence imaging (median 0.24 μM, vs. median 0.08 μM, respectively; Table 2 and Fig. 3). In prostate cancer patients injected with the old and new tracer preparation, the median concentration of intraoperatively non-detectable SNs was 5.16 and 2.44 μM, versus 1.74 and 2.86 μM for the SNs that could be detected via fluorescence imaging, respectively (Table 2 and Fig. 3).

The lowest ICG concentration in the SN of which intraoperative fluorescence detection was possible, was 0.006 μM. This was observed in a penile cancer patient.

### Pathology

In the total group, 15 patients were tumor-positive (six head-and-neck patients, five penile cancer patients, and four prostate cancer patients) with a total of 24 tumor-positive SNs.

The % ID uptake of the tumor-positive SNs on SPECT was lower compared to the non-tumor positive SNs, with a median uptake 0.26 % ID (IQR 0.13–1.04 %) and median uptake 0.86 % ID (IQR 0.24–2.08 %), respectively (Mann–Whitney U test, p-value 0.036). This, however, did not influence the intraoperative fluorescence detection rate; 83.3 % (20/24) versus 89.3 % (183/205) for tumor-postive and tumor-negative SNs, respectively (Fisher’s exact test, p-value 0.492).

## Discussion

The dose at which fluorescent dyes can be identified during surgery is an important factor for future translation and application of tracers that enable fluorescence -guided surgery. The clinical use of the hybrid tracer ICG-^99m^Tc-nanocolloid provided a unique opportunity to correlate hybrid tracer uptake in SNs based on SPECT/CT imaging measurements (^99m^Tc-label) to the intraoperative fluorescence detection rate (ICG-label). From this analysis the lower detection limit of tracer at which fluorescence guidance was still feasible could be estimated. This limit was found to be at an μM concentration level (Table 2).

SEC experiments enabled us to relate the uptake of injected dose defined on SPECT to the amount of ICG that drained in the form of ICG-^99m^Tc-nanocolloid. One critical observation herein is that the portion of non-colloidal bound ICG “stacked” in the formulation used, forming an optically silent dye deposit that could only dissolve slowly over time and after extensive washing. In this and other clinical trials using ICG-^99m^Tc-nanoclloid (current experience in over 600 patients), however, we did not observe background signal from “free” ICG, and ex vivo we found a good correlation between the presence of radioactivity and fluorescence in the SNs [11–13]. We do want to point out that SEC experiments were used to simulate and approximate the behavior of ICG-nanocolloid in human tissue in its most basic form. This experiment does not represent the full complexity of the in vivo situation, e.g. tissue heterogeneity or the presence of competing proteins, but merely showed the difference in transport for the different components present in the tracer without such effects present. Nevertheless, there appears to be a good concurrence between the basic SEC findings and the findings observed in patients.

The % ID uptake of ICG-^99m^Tc-nanocolloid in the SNs, and the volume of the SNs, allowed us to directly relate the intraoperative fluorescence imaging-based identification of SNs to the amount and/or the concentration of ICG. From this we were able to determine the lower detection fluorescence limit at which ICG provided surgical guidance. In general, when a SN is covered by (fatty) tissue fluorescence-based detection is hindered significantly [1]. To minimize the influence of this important dimension in the analysis, the surgeons involved in this study always tried to remove the overlaying tissue, prior to analyzing the fluorescent content of the SN. Obviously, this does not exclude tissue attenuation by the node itself. When fluorescence could not be used to guide the surgeon during exploration, radioguidance was used to determine the exact location of the SN.

In our study groups the median hybrid tracer uptake in the SNs was 0.82 % ID. For the SNs visualized by fluorescence imaging, the median injected dose was slightly higher, namely 0.88 % ID (IQR 0.25–2.22 % ID). For the SNs that could not be intraoperatively identified using fluorescence guidance, the median was found 0.25 % ID (IQR 0.13–0.73 % ID) (*p*-value < 0.001). In a sub-analysis comparing the uptake % ID in tumor-negative and tumor-positive SNs, the latter showed lower uptake (*p*-value 0.036), but this did not influence fluorescence imaging-based detection in vivo. These findings are in line with previous reports showing tracer uptake in SNs containing macrometastases (≤14 mm) [13].

The tracer administration could not be accurately determined in all patients. In a prospective trial this shortcoming may be compensated by using a calibrated external reference ^99m^Tc-source in the same field of view as the SNs. In this study the measured volume on the low-dose CT was used to calculate the volumes of the SNs. A more accurate calculation of the nodal volumes could be realized using, e.g. diagnostic CT or more advanced image processing/segmentation [24]. Alternatively, the weight and volume of the individual resected SNs could be defined at pathology. Unfortunately, both do not (yet) seem to be feasible in clinical routine.

The findings of the current study underline that there is a clear relation between the amount of ICG in the SNs and their detectability via fluorescence imaging in vivo. Hence, efficiency of tracer drainage identified using preoperative imaging may be predictive for the success of fluorescence-guided surgery; fluorescence examination of the surgical field should be more meticulous when the drainage is relatively low. We thus argue that the ability to assess the tracer-binding efficacy prior to surgery is a critical component in planning such interventions. It will help prevent timely and unnecessary surgical explorations towards fluorescent signals that can or cannot be detected during the operation [25].

To verify if a microdosing principle is within reach for fluorescence-based surgical guidance, one should estimate which part of the injected dosage can accumulate in the targeted lesions. A recent study of Herrmann et al. described that for the tumor-targeted tracer ^68^Ga-pentixafor, up to 0.30 % ID reached the targeted lesions [26]. If we use 0.30 % ID as representative uptake for a high affinity tracer, this would mean that intravenous administration of 100 mg [10] of a targeted tracer with a molecular weight of 3.3 kDa could yield a concentration of 0.09 μM in a lesion of 1 cm^3^. In this study ICG in SNs was detected in a concentration range of 0.006–64.61 μM with a median volume of <1 cm^3^. Combined this suggests it is legitimate to assume that a well-designed and high-affinity receptor-targeted fluorescent tracer can be applied in a microdosing principle similar to what is standard for radiotracers. The assumption that optical imaging can be effective at lower tracer doses is also in line with recent clinical reports that indicate in vivo Cerenkov imaging is feasible using standard PET-tracer formulations [27, 28]. Given the constant technical improvements that are being made in camera systems [11], one may assume that the sensitivity of fluorescence guidance will even improve further in the future. The same will be true if the signal intensity of the fluorescent dyes can be improved.

## Conclusion

For the first time we estimated the minimum amount of ICG in SNs (>0.003 nmol or >0.006 μM level) necessary to achieve a fluorescent signal suitable for intraoperative guidance. In addition our results suggest that ICG and perhaps other fluorescent-tracers can be applied on the basis of the microdosing concept.
